# Supplementary material for: Acute day units for mental health crises: a qualitative study of service user and staff views and experiences
Source: BMC Psychiatry. 2021 Mar 10;21:146. doi: 10.1186/s12888-021-03140-2 (PMC7944597; doi:10.1186/s12888-021-03140-2)
Supplement: Supplementary file 1 — Additional file 1: Appendix 1. ADU site descriptions. Appendix 2. Interview Topic Guides [file 12888_2021_3140_MOESM1_ESM.docx]

**Supplementary Information**

**Acute Day Units for mental health crises: A qualitative study of service user and staff views and experiences.**

Nicola Morant

Michael Davidson

Jane Wackett

Danielle Lamb

Vanessa Pinfold

Deb Smith

Sonia Johnson

Brynmor Lloyd-Evans

David P J Osborn

# Appendix 1: ADU site descriptions

Site descriptions are based on combined information drawn from our previous national survey of ADUs in England [13], field notes and researcher and LEAP member impressions from site visits, and other material such as operational policy documents. They are designed to provide contextual background about each service to inform readers’ understanding of findings presented in the Results. In order to protect service anonymity, pseudonyms for each service are used.

### ‘Apple’ ADU

Apple ADU has a caseload of about 25 and serves an inner-city area covering both highly deprived and wealthy areas. It is located in a freestanding building on a larger psychiatric hospital site. There is a crisis house on the same site, users of which can attend the ADU. Apple service users are more likely than users of other ADUs nationally to be from minority ethnic backgrounds, have problems with practical issues such as housing and debt, and to have diagnoses of severe and enduring psychosis. Most referrals are from acute psychiatric in-patient services. An average length of stay of 50 days was reported.

Apple ADU offered a diverse and strongly arts-based programme, including dance therapy, drama, acupuncture, aromatherapy and music-based groups. It was the only site to employ part-time arts, music and movement therapists. At the time of research, Apple ADU had operated for 15 years, making it the longest-running study site. It had evolved from a day hospital providing longer-term treatment since the early 1990s. Some staff members were long-serving, and had seen a transition from a predominantly psychodynamic approach to a more recovery-focused, short-term one. During site visits, researchers noted a sense of resistance to change among some staff members. There was a threat of closure, and loss of some ADU space during the study period which impacted negatively on staff morale. Compared to other study ADUs, researchers and LEAP members noted that Apple seemed consistently poorly attended, with sometimes only a handful of service users present. Survey data confirmed high (often over 50%) DNA rates.

###

### ‘Cherry’ ADU

Cherry ADU is situated in a commuter town close to a medium-sized city. It serves a largely rural and semi-rural catchment area that is relatively deprived compared UK norms and the other rural area ADU in the study, Lime. The service is located on a psychiatric hospital site within the main hospital building, which is grand, early-20^th^-century, and red-brick. Its interior though contrasts with this: The ADU relocated to these newly refurbished premises shortly after the study started. The unit was bright, clean and freshly painted, with service user art displayed throughout, and modern features such as a ‘relaxation room’ with large leather massage chairs and a sound system.

Cherry ADU had been in operation for seven years when data were collected. Referrals came primarily from local crisis resolution and home treatment teams (CRT). People with dementia or primary substance misuse diagnoses were not accepted, and a 30 day average length of stay was reported. The daily programme had a strongly creative focus, providing groups that were more activity-based than explicitly therapeutic or psychoeducational. The unit includes a fully-equipped medical clinic where new service users are given a full physical examination, and a weekly clozapine titration clinic is held. As users of other services attend the clozapine clinic with no additional staffing, the ADU is busier on clinic days and staff are drawn away from other ADU activities to manage this. Compared to other study sites and ADUs in England, Cherry has a smaller annual budget. Researchers became aware during site visits of significant budgetary constraints and related staff concerns. They also noted a highly dedicated staff team, some of whom raised funds for the ADU in their spare time.

###

### ‘Lime’ ADU

Lime is located in a commuter town, and like Cherry ADU, serves a largely rural catchment including small and large towns; unlike Cherry ADU, the surrounding area is relatively affluent. The ADU is situated on a larger community healthcare site in a quiet residential location. It occupies a modern purpose-built building that researchers described as spacious-feeling, well-presented and functional.

Lime ADU had been in operation for six years when data were collected. Its programme focussed primarily on psychoeducational and psychological groups. Referrals were accepted from multiple sources including the non-acute mental health sector. Lengths of stay averaged 30 days. Compared to other study ADUs, Lime reported the lowest proportion of service users experiencing psychosis, and serves more people with diagnoses of depression or anxiety than other study ADUs. People with dementia diagnoses were not accepted. Reflecting this client group, Lime was the only study ADU that did not provide a clozapine clinic, although it did include a clinic room for physical health checks. It is relatively well-funded and well-staffed, and was the only service to report in our survey that staffing felt sufficient.

### ‘Peach’ ADU

Peach ADU is located near the centre of a large and relatively affluent city. The unit is based on an expansive site, in a modern building also containing mental health in-patient facilities. As we started study recruitment Peach ADU opened for a 6-month pilot, intended to establish the feasibility of developing several larger ADUs across the local healthcare region. It was consequently much smaller than other ADUs in the study, both in terms of caseload (maximum of 10 compared to ~30 at the other sites) and physical size (comprising only three communal areas and a garden, without dedicated space for one-to-one work).

Peach ADU is jointly run, and fully integrated with the local CRT; accepts referrals only from the crisis team or inpatient wards, rather than from community teams; and all people using the ADU are also on the CRT caseload, often being treated simultaneously by both teams (for example, the CRT might do an evening home visit to deliver medication after the service user had spent the day at the ADU). Peach’s group programme consisted principally of psychoeducational and psychological groups focussed on coping strategies and symptom management. It was the only study ADU to include formal peer support worker roles. Two peer support workers attended the unit for half a day a week each in a voluntary capacity. Peach ADU closed soon after data collection for this study.

# Appendix 2: Interview Topic Guides

**AD-CARE: Qualitative study data collection**

**Semi-structured interview questions – Service users**

*Thank you for agreeing to be interviewed. As a peer researcher I’ll be interviewing you. A peer researcher is someone who has lived experience of mental health problems and using acute mental health services.*

*I would like to hear your views about this service. I’m recording this interview so I can ensure that what you have to say is accurately reflected afterwards. When we write up the findings of the study any quotes used will be anonymised so that there will be no way to identify you.*

*We will go through a series of questions in turn, but most of them are intentionally quite broad, to allow our discussions to be guided by your unique experiences. If there are any topics you feel haven’t been covered, there is space at the end for you to share any additional thoughts.*

*If there are any questions that you would rather not answer please let me know and we’ll move on. You are free to stop the interview at any time, and don’t have to give me a reason.*

**Interview questions**

1. How have you found your time using this service?
   - What were your first impressions of the service, and how have they changed over the time you’ve been here?
2. How has using the service helped you?
   - Which aspect of the service has been most helpful?
   - Which aspect of the service has been least helpful?
   - What would you change (if anything) to improve this service?
3. Have you used any other acute services (e.g. crisis team, ward, crisis house)?
   - If yes – how do you feel this service compares to that experience?
   - How would things be different for you if the service didn’t exist?
4. Can you tell me about the people here?
   - Staff?
   - Service users?
5. What are your views of the environment of this service?
   - Physical environment?
   - Atmosphere?
   - Outdoor space?
6. Do you feel safe when using this service?
   - What contributes to this feeling?
7. Is there anything else you would like to share about your experience here?

**AD-CARE: Qualitative study data collection**

**Semi-structured interview questions – Staff**

*Thank you for agreeing to be interviewed.*

*I would like to hear your views about this service. I’m recording this interview so I can ensure that what you have to say is accurately reflected afterwards. When we write up the findings of the study any quotes used will be anonymised so that there will be no way to identify you.*

*We will go through a series of questions in turn, but most of them are intentionally quite broad, to allow our discussions to be guided by your unique experiences. If there are any topics you feel haven’t been covered, there is space at the end for you to share any additional thoughts.*

*If there are any questions that you would rather not answer please let me know and we’ll move on. You are free to stop the interview at any time, and don’t have to give me a reason.*

**Interview questions**

1. What’s it like working here?
   1. How (if at all) has the service changed since you have worked here?
2. How do you feel the service helps people?
   1. Which aspect of the service work well?
   2. Which aspect of the service work less well?
   3. What would you change (if anything) to improve this service?
   4. For carers?
3. Do you have any experience of working in other acute services (e.g. crisis team, ward, crisis house)?
   1. How do you feel this service compares to other acute services?
   2. How would things be different if the service didn’t exist?
4. Can you tell me about the people here?
   1. Staff/service users/carers?
5. What are the most rewarding aspects of your role?
6. What are the most challenging aspects of your role?
7. What are your views of the environment of this service?
   1. Physical environment?
   2. Atmosphere?
   3. Outdoor space?
8. Do people feel safe here?
   1. What contributes to this feeling?
   2. Staff?
9. Is there anything else you would like to share about your experience here?

**AD-CARE: Qualitative study data collection**

**Semi-structured interview questions – Carers**

*Thank you for agreeing to be interviewed.*

*I would like to hear your views about this service. I’m recording this interview so I can ensure that what you have to say is accurately reflected afterwards. When we write up the findings of the study any quotes used will be anonymised so that there will be no way to identify you.*

*We will go through a series of questions in turn, but most of them are intentionally quite broad, to allow our discussions to be guided by your unique experiences. If there are any topics you feel haven’t been covered, there is space at the end for you to share any additional thoughts.*

*If there are any questions that you would rather not answer please let me know and we’ll move on. You are free to stop the interview at any time, and don’t have to give me a reason.*

**Interview questions**

1. As someone who provides support for X [service user], what’s your experience of this service been like?
   1. What were your first impressions of the service, and how have they changed over time?
2. How do you feel the service has helped?
   1. Which aspect of the service has been most helpful?
   2. Which aspect of the service has been least helpful?
   3. What would you change (if anything) to improve this service?
   4. For you/the person you care for? (If either not covered already)
3. Do you have any experience of other acute services as X’s carer / supporter (e.g. crisis team, ward, crisis house)?
   1. If yes – how do you feel this service compares to that experience?
   2. How would things be different if the service didn’t exist?
4. Can you tell me about the people here?
   1. Staff?
   2. Other service users?
5. What are your views of the environment of this service?
   1. Physical environment?
   2. Atmosphere?
   3. Outdoor space?
6. When you have visited the service, did you feel safe there?
   1. What contributed to this feeling?
7. Has the service provided any support specifically for you as a carer?
8. Is there anything else you would like to share about your experience here?
